# Novel insights into immunopathogenesis and crucial biomarkers between primary open‐angle glaucoma and systemic lupus erythematosus

**DOI:** 10.1002/imo2.27

**Published:** 2024-09-10

**Authors:** Yixian Liu, Mengling You, Zhou Zeng, Jing Wang, Rong Rong, Xiaobo Xia

**Affiliations:** ^1^ Eye Center of Xiangya Hospital Central South University Changsha China; ^2^ Hunan Key Laboratory of Ophthalmology Changsha China; ^3^ National Clinical Key Specialty of Ophthalmology Changsha China; ^4^ National Clinical Research Center for Geriatric Diseases (Xiangya Hospital) Central South University Changsha China

**Keywords:** immune infiltration, molecular docking, primary open‐angle glaucoma, single cell transcriptome, systemic lupus erythematosus, targeted drugs

## Abstract

Glaucoma is the primary factor underlying irreversible blindness. Recent studies suggest that the risk of glaucoma significantly increases in patients with systemic lupus erythematosus (SLE); however, the mechanism underlying this association remains unclear. Therefore, this study aimed to identify novel common biomarkers and potential therapeutic drugs for SLE and glaucoma. GSE27276, GSE50772, and GSE148371 datasets were sourced from the gene expression omnibus (GEO) database. An integrated analysis of the datasets for both diseases identified biomarkers and thoroughly examined their biological roles and molecular mechanisms using differential expression analysis (DEA), weighted gene co‐expression network analysis (WGCNA), gene enrichment analysis, machine learning, microRNA (miRNA) and transcription factor analyses, immune infiltration analyses, and single‐cell transcriptome analysis. Concurrently, molecular docking was used to forecast potential drugs targeting these biomarkers. Finally, reverse transcription quantitative real‐time polymerase chain reaction (RT‐qPCR) was performed in human trabecular meshwork stem cells to validate the five identified biomarkers. The 10 key genes identified through DEA and WGCNA were predominantly involved in immune, inflammatory, and autophagy pathways. Additionally, machine learning identified five biomarkers, and we established associated transcription factors and miRNA regulatory networks. Immune infiltration analysis indicated an elevated presence of immune cells, including macrophages, T cells, and B cells, in both conditions. Furthermore, the DSigDB database yielded 10 potential therapeutic agents, three of which showed strong binding potential to the biomarkers via molecular docking. The RT‐qPCR results confirmed the trend in gene expression. This study uncovered a new link between SLE and primary open‐angle glaucoma, identifying biomarkers and mechanisms of immunopathogenesis for future research and treatment strategies.

## Highlights

- We first explored the relationship between systemic lupus erythematosus (SLE) and primary open‐angle glaucoma (POAG) based on gene transcriptome analysis and machine learning.
- Association of the pathogenesis of SLE‐related POAG and five biomarkers with immune cells assessed by immune infiltration analysis and single‐cell transcriptome analysis.
- A regulatory network involving biomarker‐based transcription factors and miRNAs was constructed.
- We predicted targeted drugs based on crucial biomarkers and analyzed the potential clinical value of these small molecule drugs.

## Graphical abstract

This study revealed a novel correlation between systemic lupus erythematosus (SLE) and primary open‐angle glaucoma (POAG) through comprehensive bioinformatics analysis and experimentation, identifying biomarkers and immunopathogenesis for future research and treatment of SLE‐related POAG.

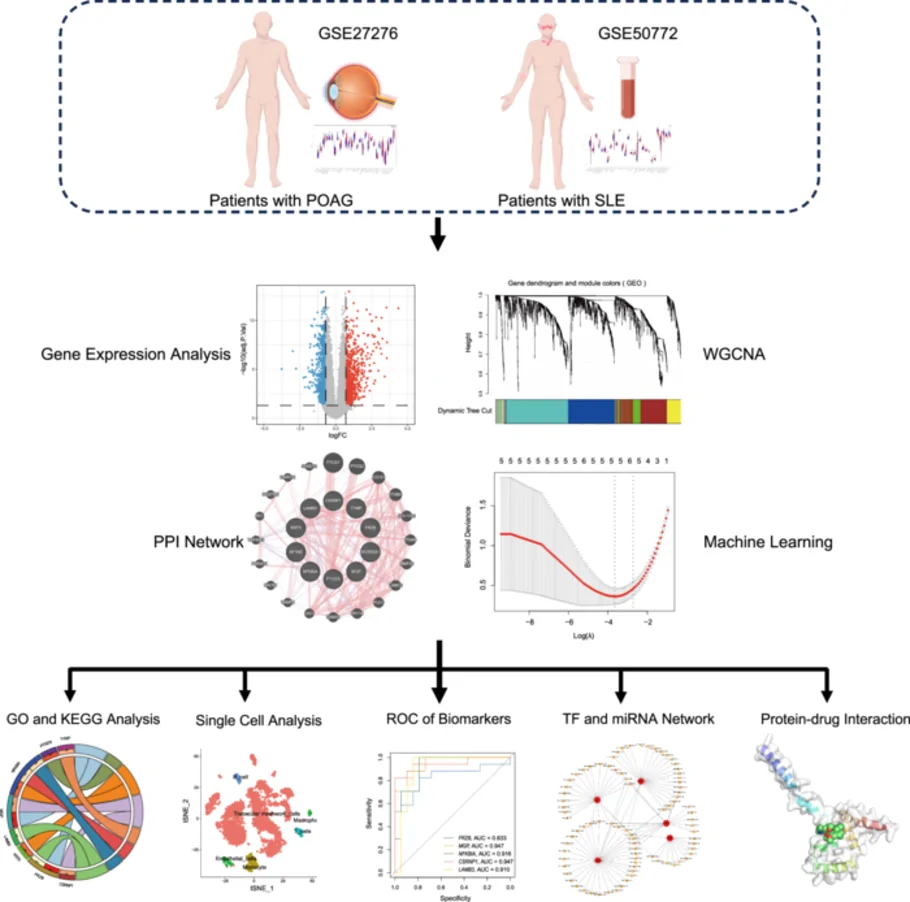

## 1 INTRODUCTION

As the foremost cause of irrevocable blindness globally, glaucoma is distinguished by the gradual loss of retinal ganglion cells (RGCs) owing to damaged neuronal axons, and pathologically high intraocular pressure (IOP) has been acknowledged as a significant risk determinant. According to epidemiological projections, approximately 112 million individuals worldwide are projected to develop glaucoma by 2040 [1, 2]. Glaucoma is categorized into three main types: primary, secondary, and congenital. Primary open‐angle glaucoma (POAG) constitutes approximately 70% of all patients with glaucoma [3]. While the etiology of glaucoma is complex and multifactorial, it has been shown to include abnormalities in the anatomy of the eye, obstruction of the aqueous drainage system, secondary ocular trauma, and systemic diseases. The trabecular meshwork is an important pathway for aqueous outflow from the atrium and has a significant impact on maintaining IOP stabilization [4]. As such, abnormalities in the morphology, structure, and function of the trabecular meshwork are all key pathogenic mechanisms in POAG.

A variety of immune cells have been closely associated with the progression of neurodegenerative diseases, including multiple sclerosis and Alzheimer's disease [5]. Recent studies have further identified the immune response as a crucial aspect of the pathophysiology of POAG [6]. Under physiological conditions, the blood‐retinal barrier (BRB) of the eye prevents the infiltration of pathogens and immune cells, but a pathologically high IOP can cause damage to the BRB, causing an influx of immune cells in the retina [7]. Furthermore, research has revealed that the existence of autoantibodies targeting heat shock proteins in glaucoma can lead to optic nerve degeneration and death of RGCs [5]. Evidence has also indicated changes in the concentration of autoantibodies against retinal and optic nerve head antigens among individuals with glaucoma. This alteration in physiological balance can lead to a shift in the immune system from regulatory immunity to inflammatory and neurodegenerative processes that drive glaucoma progression [8]. Systemic lupus erythematosus (SLE) is also intricately linked with immune factors, impacting multiple systems. Its pathogenesis is closely related to T lymphocyte dysfunction and abnormal activation of B lymphocytes, ultimately leading to the production of autoantibodies that cause an abnormal immune response and affect the function of multiple organs and systems [9]. Although SLE is a chronic autoimmune disease that can cause multiorgan damage, many patients can achieve good disease control and a high quality of life through appropriate treatment and long‐term management. Approximately one‐third of patients with SLE show ocular involvement, which may include severe unilateral or bilateral vision loss of varying degrees [10]. In a 6‐year national cohort study, researchers found that the relative risk (RR) for the incidence of glaucoma in patients with SLE was 9.01, indicating a strong association between the two diseases [11]. In addition, emerging evidence suggests that the risk of glaucoma is significantly heightened in patients with SLE, compared with that in those without the condition [12, 13]. This also suggests that there may be common pathogenic mechanisms between SLE and POAG.

Long‐term glucocorticoid use has been shown to cause glucocorticoid‐induced glaucoma in patients with SLE [14]. However, if there are common mechanisms underlying SLE and glaucoma, such as immune‐related factors, they have not yet been demonstrated. With the development of bioinformatic techniques, gene‐level‐based studies of these two diseases could provide new perspectives on their common etiology and pathogenesis. As previously mentioned, POAG is the most common and representative type of glaucoma. Therefore, in the present study, we performed extensive bioinformatics analyses of gene expression data obtained from patients with POAG and SLE to identify common pathways and hub genes mediating the two diseases, as well as to explore potential targeted therapeutic agents against these genes. Our findings strongly indicate that these genes may wield significant regulatory influence over both diseases, laying a scientific foundation for future investigations into the immunopathogenesis of POAG and potential new options for the clinical treatment of SLE‐related POAG.

## 2 RESULTS

### Screening of key genes in POAG and SLE

**Figure 1:**
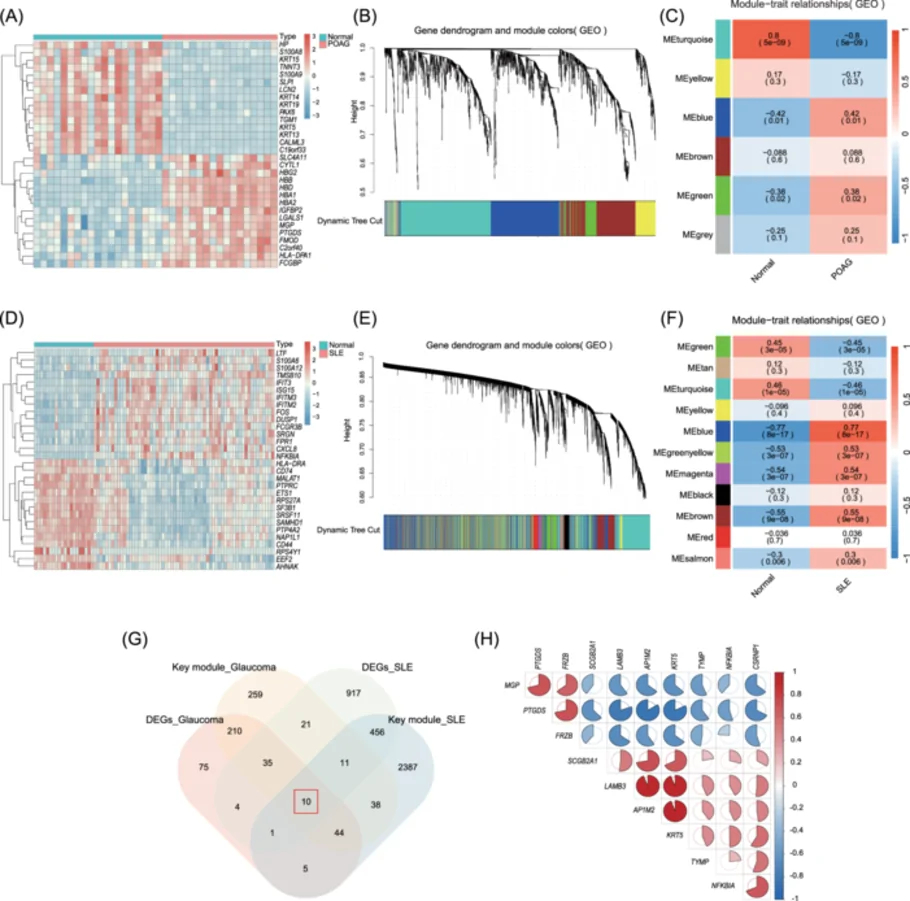
Differentially expressed genes (DEGs) and weighted gene co‐expression network analysis (WGCNA) of systemic lupus erythematosus (SLE) and primary open‐angle glaucoma (POAG) datasets. (A) Heatmap of DEGs in POAG. Colors indicate changes in gene expression levels, with red indicating upregulation and blue indicating downregulation. (B) Clustering dendrogram of genes in POAG, different colors represent different gene modules. (C) Relationship graph between modules and POAG. (D) Heatmap of DEGs in SLE. (E) Clustering dendrogram of genes in SLE, different colors represent different gene modules. (F) Relationship graph between modules and SLE. (G) Venn diagram of the intersection of DEGs and genes obtained from WGCNA. (H) Correlation heatmap of the 10 crucial genes.

We investigated the RNA‐seq data of human trabecular mesh tissues acquired from 17 patients with POAG and 19 controls, in addition to RNA‐seq data collected from 61 SLE patients and 20 healthy controls (Figure 1A,D, Tables S1 and S2). Furthermore, we screened differentially expressed genes (DEGs) in POAG and SLE using differential expression analysis, with 384 DEGs in the POAG data set and 1455 DEGs in the SLE data set (Figure S1).

Weighted gene co‐expression network analysis (WGCNA) was employed to construct gene co‐expression networks, aiming to discern the most relevant gene modules specific to each disease. Six modules were obtained from the co‐expression network constructed in the POAG samples (Figure 1B,C), among which the MEturquoise module, comprising 628 genes, demonstrated the strongest correlation with POAG (Cor = 0.72, *p* = 2.1 × 10^−^^101^) (Figure S2A). Furthermore, 11 modules were obtained from the co‐expression network constructed in the SLE samples (Figure 1E,F), among which the MEblue module, containing 2952 genes, demonstrated the strongest correlation with SLE (Cor = .87, *p* = 1 × 10^−^^200^) (Figure S2B). Finally, 10 key genes were identified by intersecting the acquired genes with the overlapping DEGs (Figure 1G). Furthermore, a correlation heatmap analysis on the selected crucial genes revealed strong correlations among the 10 genes (Figure 1H).

### Pathway and functional analysis of key genes

**Figure 2:**
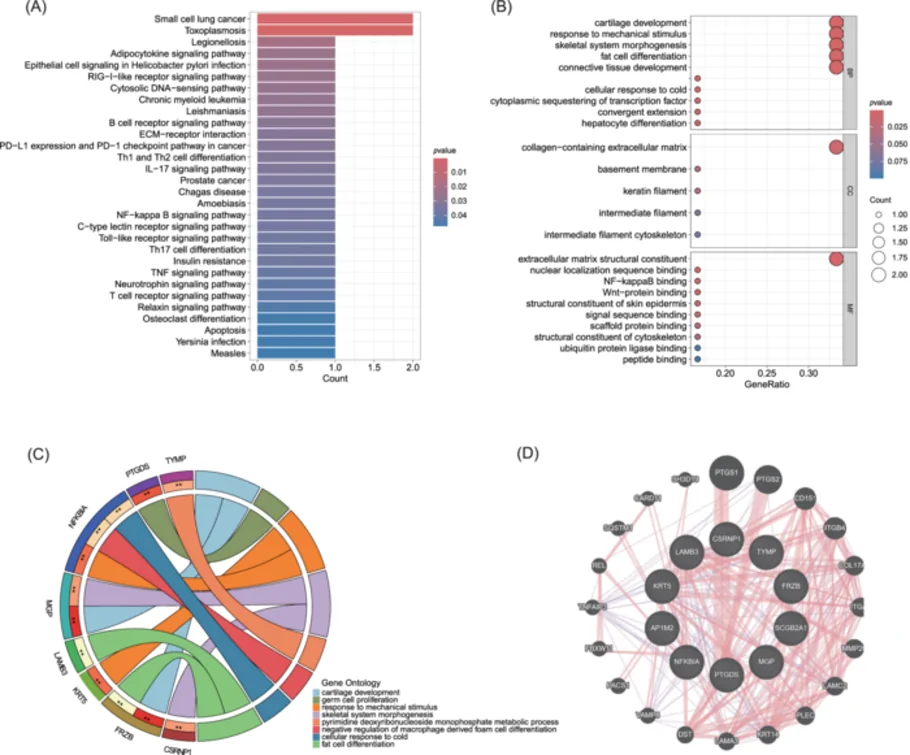
Gene and pathway enrichment analysis of 10 key genes. (A) Bar graph of Kyoto Encyclopedia of Genes and Genomes (KEGG) enrichment analysis. (B) Bubble graph of gene ontology (GO) enrichment analysis. (C) Chordal graph of GO enrichment analysis, indicating the eight significantly enriched GO entries and corresponding genes. (D) Network interaction map of GeneMANIA analysis.

To delve deeper into the underlying biological functions of these crucial genes, the Kyoto Encyclopedia of Genes and Genomes (KEGG) and Gene Ontology (GO) enrichment analyses were conducted. KEGG enrichment analysis demonstrated that these crucial genes were predominantly enriched in small‐cell lung cancer, PD‐1/PD‐1L, B and Th cells, and the Toll‐like receptor pathway (Figures 2A and S3B), while GO enrichment analysis demonstrated that these crucial genes were primarily enriched in macrophage and fat cell differentiation and the NF‐κB and Wnt‐protein pathways (Figures 2B,C and S3A). In addition, GeneMANIA was used to construct gene networks interacting with key genes. We observed that these interacting genes were predominantly associated with inflammatory pathways, such as prostaglandin biometabolism and synthesis (Figure 2D).

### Screening and validation of hub genes based on machine learning and experiment

**Figure 3:**
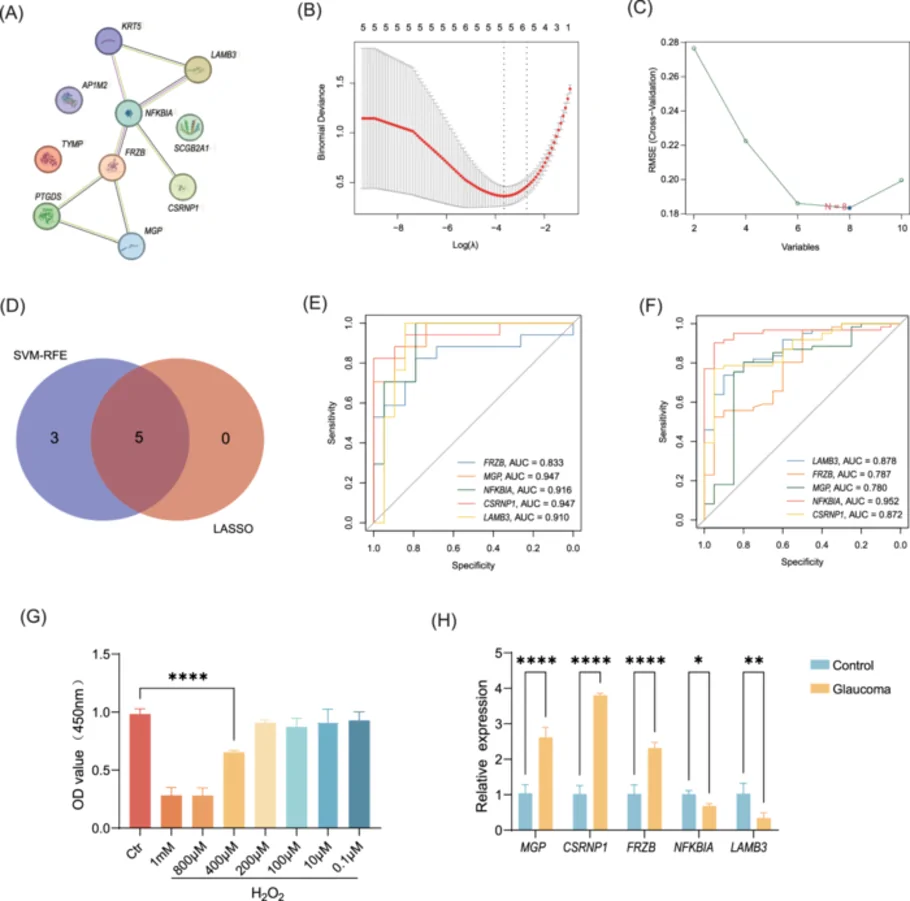
Screening and validation of candidate diagnostic biomarkers in patients with systemic lupus erythematosus (SLE)‐related primary open‐angle glaucoma (POAG). (A) Protein–protein interaction (PPI) network construction. (B) Cross‐validation curve of least absolute shrinkage and selection operator (LASSO) regression analysis, the dashed line on the left side corresponds to the position of the minimum error with a horizontal coordinate variable of five. (C) Biomarkers screened using the support vector machine recursive feature elimination (support vector machine recursive feature elimination [SVM‐RFE]) algorithm. (D) Venn plots show the five diagnostic biomarkers common to LASSO and SVM‐RFE. (E, F) Receiver operating characteristic (ROC) curves of the diagnostic biomarkers in the SLE and POAG datasets. (G) Indicates the cell viability of trabecular meshwork stem cells at different concentrations of H_2_O_2_. (H) The mRNA levels of *MGP*, *CSRNP1*, *FRZB*, *NFKBIA*, and *LAMB3*. *p*‐Values of <0.05, <0.01, and <0.0001 were indicated by the symbols *, **, and ****, respectively.

First, a protein–protein interaction (PPI) network for these 10 crucial genes revealed strong correlations among seven of them (Figure 3A). To further explore potential diagnostic biomarkers in patients with SLE‐related POAG, least absolute shrinkage and selection operator (LASSO) regression analysis was applied based on lambda function (Figure 3B) and identified five hub genes (*LAMB3*, *MGP*, *NFKBIA*, *CSRNP1*, and *FRZB*). Eight hub genes (*KRT5, PTGDS*, *CSRNP1*, *LAMB3*, *NFKBIA*, *MGP*, *AP1M2*, and *FRZB*) were further identified through the support vector machine recursive feature elimination (SVM‐RFE) algorithm (Figure 3C). Subsequently, the intersecting genes in these two groups were identified to obtain five hub genes (Figure 3D), including two upregulated genes (*MGP* and *FRZB*) and three downregulated genes (*LAMB3*, *CSRNP1*, and *NFKBIA*). The area under the curve (AUC) values of these five biomarkers performed well in POAG and SLE (Figure 3E,F), indicating that these genes were well represented in patients with SLE‐related POAG.

Furthermore, we explored the modeling concentration of trabecular meshwork stem cells in the in vitro glaucoma model using CCK‐8. A decrease in cell viability of approximately 50% was observed after treatment with 400 μm H_2_O_2_, indicating this as the optimal modeling concentration (Figure 3G). Subsequently, reverse transcription quantitative real‐time polymerase chain reaction (RT‐qPCR) validated the expression changes of these biomarkers, and the findings illustrated that the expressions of *MGP*, *CSRNP1*, and *FRZB* were elevated, with *CSRNP1* and *MGP* being more significantly elevated, whereas the expressions of *NFKBIA* and *LAMB3* were decreased. Among these, *CSRNP1* was predicted to be downregulated in the trabecular meshworks of patients with POAG; however, RT‐qPCR verified an upregulation (Figure 3H).

In this context, to elucidate how microRNA (miRNA) and transcription factors (TFs) regulate key genes at the transcriptional level, network analysis of miRNA and TF interactions with hub genes was performed and visualized using Cytoscape. In the miRNA regulatory network, a total of 84 miRNAs interacted with *MGP*, *LAMB3*, *NFKBIA*, *CSRNP1*, and *FRZB* (Figure S4A). In the TF regulatory network, 34 TFs interacted with five hub genes (Figure S4B). Four of these TFs (*FOXC1*, *ARID3A*, *NFKB1*, and *TFAP2A*) interacted with three biomarkers. Three miRNAs (hsa‐mir‐124‐3p, hsa‐mir‐1‐3p, and hsa‐mir‐23b‐3p) interacted with four biomarkers, and four miRNAs (hsa‐mir‐27a‐3p, hsa‐mir‐27a‐5p, hsa‐mir‐191‐5p, and hsa‐mir‐21‐3p) interacted with three biomarkers, suggesting that these miRNAs and TFs may have close interactions with these biomarkers. Therefore, the miRNA and TF regulatory network establishes a theoretical foundation for revealing potentially actioning signaling networks based on these biomarkers.

### Immune infiltration analysis identified two diseases and hub genes linked to immune cells

**Figure 4:**
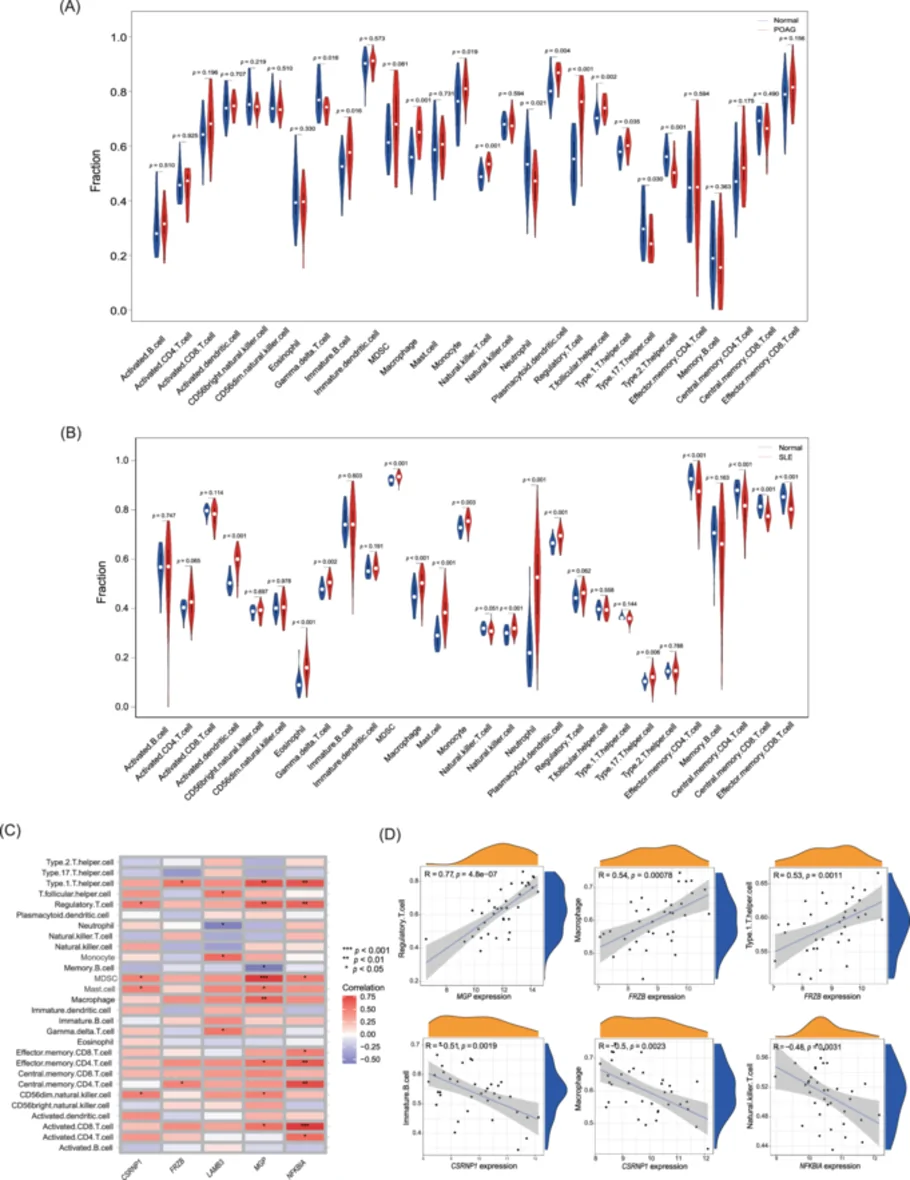
Immune infiltration analysis. (A) Variation in the relative abundance of infiltrating immune cells in patients with primary open‐angle glaucoma (POAG) versus that in healthy controls. (B) Variation in the relative abundance of infiltrating immune cells in patients with systemic lupus erythematosus (SLE) versus that in healthy controls. (C) Correlation analysis between biomarkers and immune cells. (D) Scatter plot of the association between biomarkers and immune cells.

As immune‐inflammatory activity was found to be high and a key pathogenic factor in both diseases, we performed an immune infiltration analysis of disease data from patients with POAG and SLE, finding that B cells, macrophages, monocytes, and regulatory T‐cell phase infiltration abundance were highly increased in patients with POAG, compared with those in controls (Figure 4A). Additionally, Th cells, regulatory T cells, macrophages, mast cells, and neutrophils were found to have a higher infiltration abundance in patients with SLE (Figure 4B).

To build a deeper understanding of the connection between biomarkers and immune cells, immune infiltration analysis of these five biomarkers showed strong correlations with Th17 cells, regulatory T cells, CD8^+^ T cells, macrophages, and B cells (Figure 4C,D). These results suggest that the biomarkers likely participate in the regulation of immune‐related biological activities.

### Prediction and molecular docking of potential target drugs

**Figure 5:**
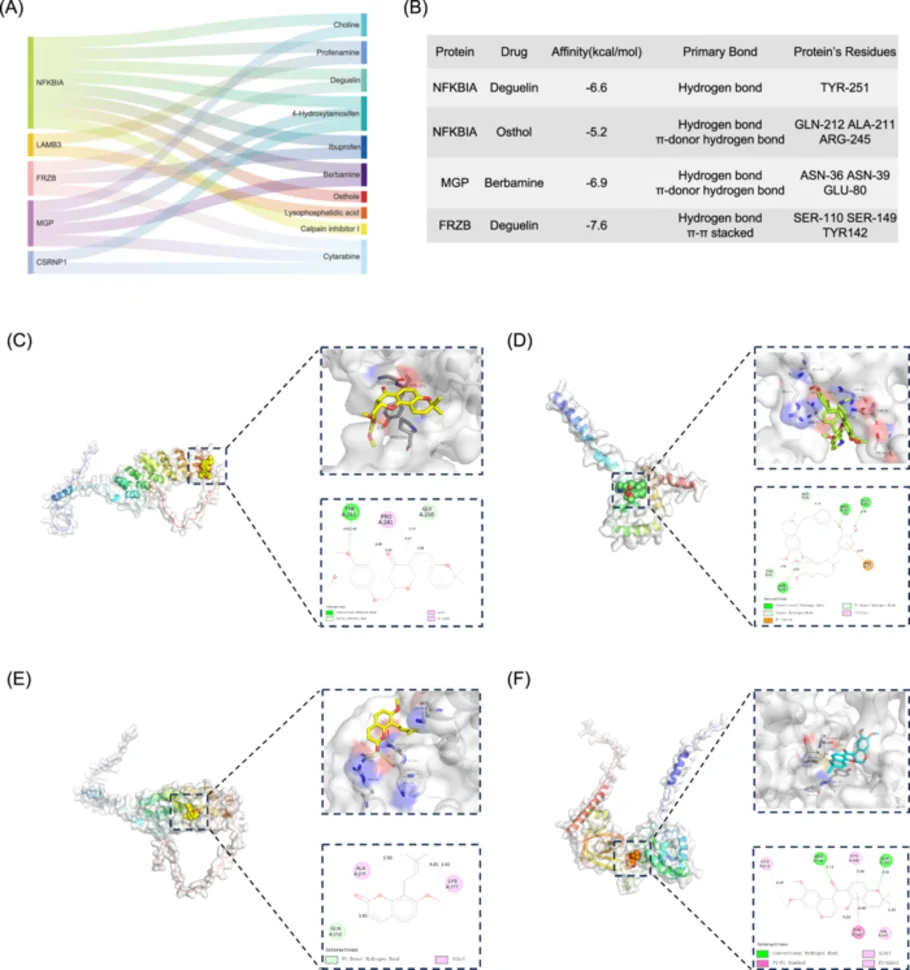
Prediction of candidate drugs. (A) Sankey diagram of hub genes and potential drugs. (B) Molecular docking results of drugs with targets. (C) Interactions between the *NFKBIA* and deguelin protein‐ligand complex in 3D and 2D diagrams. (D) Interactions between the *MGP* and berbamine protein‐ligand complex in 3D and 2D diagrams. (E) Interactions between the *NFKBIA* and osthole protein‐ligand complex in 3D and 2D diagrams. (F) Interactions between the *FRZB* and deguelin protein‐ligand complex in 3D and 2D diagrams.

Previously, we confirmed the potential regulatory roles of the aforementioned biomarkers in both POAG and SLE. To further explore any potential effective drugs targeting these genes, we obtained 10 potential therapeutic agents from the DSigDB database based on *p*‐values (Figure 5A). Among these 10 drugs, choline, deguelin, berbamine, lysophosphatidic acid, and osthole have been extensively researched and explored in the context of glaucoma, as well as other neurodegenerative diseases or autoimmune diseases, such as rheumatoid arthritis, suggesting that these drugs have high potential as effective clinical therapeutic candidates for glaucoma. Therefore, we further conducted molecular docking of these five small molecule drugs with their predicted targets. Based on the formation of hydrogen bonds and affinities of <−5 kcal/mol, we selected the best four drug‐protein complexes for visualization (Figure 5B) and displayed the binding sites and interaction forces between the drugs and proteins. Using molecular docking technology, we identified the binding sites of these drugs with biomarkers and demonstrated their potential effectiveness and prospects for development as treatments for glaucoma (Figure 5C–F).

### Expression and distribution of biomarkers in single cells

**Figure 6:**
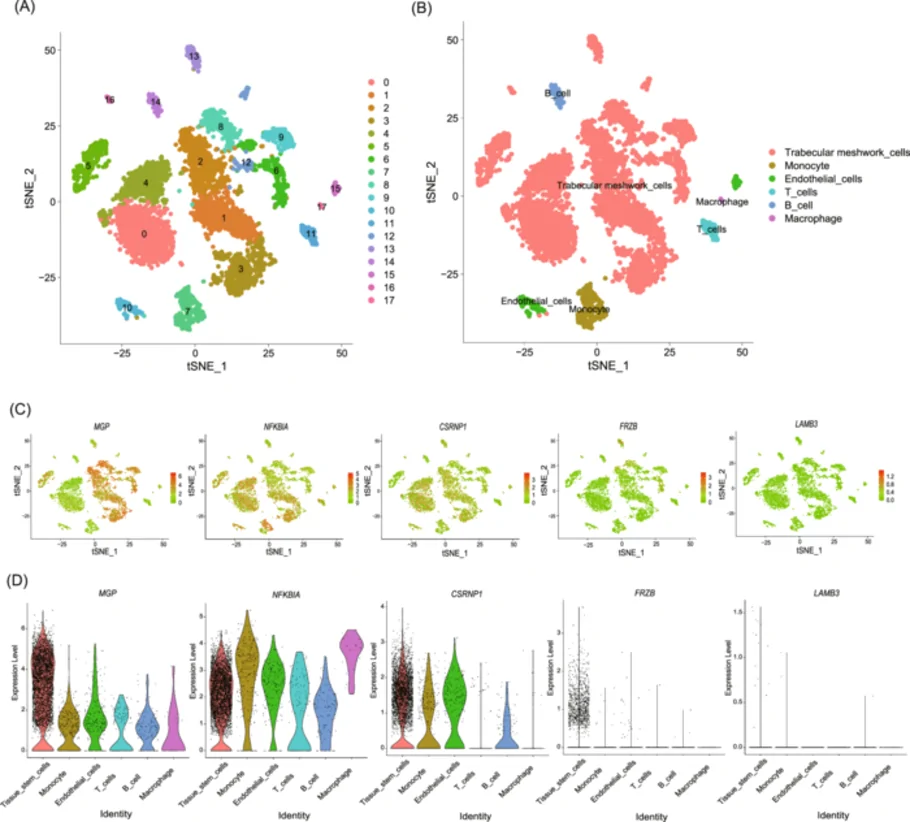
Expression and distribution of hub genes in single cells of trabecular meshwork tissues. (A, B) Cell types of trabecular meshwork tissues in patients with glaucoma. (C, D) Scatter and violin plots of the five hub genes.

To investigate the specific expression and distribution of biomarkers within the trabecular meshwork tissue of patients with glaucoma, we initially clustered the trabecular meshwork cells into 17 categories by analyzing trabecular meshwork single‐cell transcriptome data (Figure 6A), which were further annotated into six different cells (Figure 6B). Furthermore, we investigated the expression of the five biomarkers in various cells, with results showing that *MGP*, *CSRNP1*, *FRZB*, and *NFKBIA* were highly expressed in trabecular meshwork stem cells; *NFKBIA*, *MGP*, and *CSRNP1* were abundantly expressed in endothelial cells, monocytes, macrophages, T cells, and B cells; and *LAMB3* was less expressed in trabecular meshwork tissues (Figure 6C,D). These results indicate that the biomarkers play significant regulatory roles in trabecular meshwork and immune cells in trabecular meshwork tissues.

## 3 DISCUSSION

The pathogenesis of POAG is complex, with high IOP recognized as a significant pathogenic factor. The formation of IOP is closely related to aqueous humor circulation, and the trabecular meshwork constitutes the primary component of the aqueous humor outflow pathway, playing a pivotal role in maintaining normal IOP. Oxidative stress directly contributes to high IOP in glaucoma and may lead to the accumulation of the extracellular matrix and degenerative changes, resulting in trabecular meshwork cell dysfunction. This process ultimately leads to an increased resistance to aqueous humor outflow and pathologic IOP elevation. In recent years, increasing evidence has shown that inflammation and the inflammatory response can mediate the development of POAG and that the inflammatory response and oxidative stress are typically closely related, although their potential mechanisms require further exploration. As an autoimmune disease with multisystem involvement, immune‐inflammatory activities play a crucial role in SLE. The pathological process of SLE mainly includes the abnormal activation of the immune system and the production of autoantibodies, these autoantibodies immune complex formation, sedimentary in multiple organs and tissues, causing inflammation and tissue damage. With increasing research on the disease, several studies have found that individuals with SLE are at a substantially heightened risk of developing glaucoma [11, 12, 13]. The long‐term use of glucocorticoids in patients with SLE has further been shown to cause glucocorticoid‐induced glaucoma. Because the pathogenesis of both SLE and POAG are closely related to immune and inflammatory factors, whether common pathogenic factors other than glucocorticoid exist between the two diseases currently remains unclear.

To explore a deeper link between the pathogenesis of SLE and POAG, we explored the disease‐related genes and pathways common to patients with both diseases using bioinformatic methods, additionally laying a scientific foundation for future research of the immunopathogenesis associated with POAG. The key genes in these two diseases were mainly enriched in the immune cell pathways of macrophage differentiation, B cells, and T cells. Prior research has shown that the activation and infiltration of a significant number of macrophages were observable in the optic nerve papilla region of patients with glaucoma [15]. Activated macrophages can generate pro‐inflammatory factors, such as TNF, IL‐12, and IL‐18, and induce T‐cell‐mediated immune responses [16]. Researchers have simultaneously found a large infiltration of T cells in the retina, which is pivotal in the loss of RGCs in patients with glaucoma [17]. The dysfunction and abnormal activation of B and T cells are also important pathogenic mechanisms in SLE. These crucial genes were enriched in Toll signaling, apoptosis, and *PD‐1/PD‐L1* pathways. The Toll signaling pathway holds significant importance in the pathogenesis of POAG, inducing trabecular meshwork cell fibrosis by regulating TGF‐β2 and inducing apoptosis and inflammatory responses in RGCs via the caspase pathway and inflammatory factors [18, 19, 20]. Furthermore, activated T and B cells are the primary cells expressing *PD‐1/PD‐L1* [21], with the results revealing that the number of RGCs in a mouse model of chronic ocular hypertension with reduced *PD‐1/PD‐L1* expression was greater than that in the non‐intervention group. This expression could regulate the process of cellular inflammation and apoptosis by mediating signaling pathways, such as TNF, NF‐κB, and *PI3K/Akt*, and exerting neuroprotective effects [22, 23]. These findings indicate that POAG and SLE share several common pathogenic mechanisms, such as inflammatory response pathways.

Using machine learning algorithms, five genes (*LAMB3*, *MGP*, *NFKBIA*, *CSRNP1*, and *FRZB*) were identified as the hub signature genes of POAG and SLE, which are predominantly associated with immune and inflammatory pathways. Among them, *LAMB3*, *CSNP1*, and *FRZB* were first identified in glaucoma and SLE. Moreover, a series of machine learning algorithms also indicated that these biomarkers possess high diagnostic value for the disease. Further, the Gla protein encoded by *MGP* is widely expressed in trabecular meshwork cells, where *MGP* mediates the calcification process of trabecular meshwork tissues by regulating ALP activity and calcium metabolism. This results in increased stiffness and decreased compliance with the trabecular meshwork, which in turn affects atrial fluid drainage, leading to high IOP [24]. Patients with SLE have recently been shown to suffer from endoplasmic reticulum stress owing to abnormalities in the calcium metabolic pathways that mediate the dysregulation of immune cells, such as B cells and T cells. Therefore, we speculated that MGP might also exert a regulatory role in this process [25, 26]. The *Wnt* signaling pathway is crucial for controlling the levels of cellular apoptosis and inflammation [27, 28], and research has shown that this pathway can be targeted to repair brain injury caused by neuroinflammation [29]. Further studies have also supported its pivotal role in regulating the actin skeleton of the trabecular meshwork [30, 31]. In recent years, the promising role of *Wnt* signaling pathway inhibitors (DKK‐1) in treating autoimmune diseases like SLE has attracted widespread attention [32]. Both *FRZB* and *CSRNP1* act as significant regulators of the Wnt signaling pathway [33, 34], and we speculated that they may also target the *Wnt* signaling pathway to fulfill significant regulatory roles in glaucoma and SLE. Although the expression of *CSRNP1* in our experimental group did not align with our hypothesis, it has been shown to be significantly elevated in some studies of inflammatory and neurodegenerative diseases, where it functions to mediate apoptosis and inflammatory responses by triggering the *Wnt/β‐catenin* pathway [35, 36, 37]. This is consistent with the trend we experimentally verified in the present study.

The *NFKBIA* gene encodes IKB‐α, belonging to the NF‐κB inhibitor family, which reduces inflammatory responses by restraining the activity of the NF‐κB/REL complex [38]. NF‐κB is a key regulatory molecule in immune and inflammatory responses [39, 40]. Research has indicated that NF‐κB can mediate trabecular meshwork injury and apoptosis and neuroinflammation in RGCs by regulating the expression of inflammatory mediators, such as TNF‐α and IL‐1, and oxidative stress [41, 42, 43]. This pathway is intricately linked to the inflammatory response and disease progression in patients with SLE [44]. *LAMB3* has the capacity to induce cell cycle arrest and apoptosis by modulating the *PI3K/Akt* pathway [45], and *PI3K/Akt* has been shown to assume pivotal roles in both the oxidative stress response and apoptosis in RGCs and trabecular meshwork cells [46, 47] and in modulating the activation and growth of T and B cells in patients with SLE [48, 49].

MiRNAs and TFs also exert significant roles in regulating gene expression. In the current study, we developed miRNA and TF regulatory networks. In the miRNA regulatory network, three miRNAs, hsa‐mir‐124‐3p, hsa‐mir‐1‐3p, and hsa‐mir‐23b‐3p, were found to be most closely associated with the biomarkers. Studies have shown that hsa‐mir‐124 can regulate cell death, autophagy, mitochondrial dysfunction, and the inflammatory response [50, 51]. In the TF regulatory network, the TFs *FOXC1*, *NFKB1*, and *TFAP2A* play important roles in mediating immune‐inflammatory activities and oxidative stress [52, 53].

Targeted drugs based on hub gene prediction can also provide a new scientific basis and direction for future clinical treatments. Through molecular docking, we discovered that three drugs have low binding affinities with biomarkers but exhibit strong interactions with each other, suggesting their potential to target the biomarkers. These drugs are also extensively studied in retinal and neurodegenerative diseases. Studies have shown that osthole, a coumarin derivative extracted from serpentine, is a potential *AMPK* agonist that modulates inflammatory activity in rheumatoid arthritis by suppressing *NLRP3* inflammatory vesicle activation through modulation of mitochondrial homeostasis [54]. In addition, recent evidence has suggested that osthole can exert neuroprotective effects in some neurodegenerative diseases by stimulating signaling pathways, such as the *Notch*, *BDNF/Trk*, and *P13k/Akt* pathways [55, 56]. Deguelin, a naturally occurring carotenoid compound found in leguminous plants, has demonstrated efficacy in inhibiting retinal neovascularization [57] while also exerting anti‐inflammatory activity through inhibition of the NF‐κB pathway [58]. Berbamine, a natural compound derived from the traditional Chinese medicine Phellodendron Bark, has exhibited anti‐inflammatory effects through inhibition of COX‐2 and NF‐κB and has further been demonstrated to ameliorate the neuroinflammation and neurodegenerative pathologies induced by diabetes [59]. Berbamine also reduces inflammation and apoptosis in diabetic retinopathy. However, we have only predicted the effectiveness of these drugs from a computational perspective; further experimental and clinical research will be needed to demonstrate their actual therapeutic effects in clinical settings. Nevertheless, this discovery provides an important direction for future glaucoma drug development and disease treatment.

The immunopathogenesis of glaucoma has also been proposed in recent years, and additional studies are needed to explore its association with immune cells. In this study, the correlation of POAG and SLE data samples with immune cells and the correlation between common biomarkers and immune cells were explored through a series of bioinformatics analyses. In immune infiltration analysis, patients with POAG and SLE were closely associated with immune cells, such as macrophages, B cells, and T cells. The expression and distribution of five genes in glaucomatous trabecular meshwork tissues were simultaneously explored using single‐cell transcriptome analysis, which found that *MGP, NFKBIA, CSRNP1*, and *FRZB* were abundantly distributed in trabecular meshwork stem cells, indicating that these biomarkers are pivotal in POAG mediated by trabecular meshwork cell abnormalities. Moreover, these hub genes were also abundantly expressed and distributed in monocytes, endothelial cells, B cells, and T cells in the trabecular meshwork tissues, consistent with the pathway and immune infiltration analyses performed previously. Based on these studies, we hypothesized that these biomarkers exert significant regulatory roles in trabecular meshwork cells and exert important synergistic roles through actions of immune cells, such as T cells, B cells, and monocytes in the trabecular meshwork tissues, thus jointly mediating POAG.

Finally, this study has some limitations. Most of the study is based on bioinformatics analyses and predictions, and we have not corroborated our predictions with extensive experiments. In future studies, we plan to investigate key biomarkers and potential drugs to improve preclinical applications.

## 4 CONCLUSIONS

This study established an association between glaucoma and SLE through preliminary biochemical analysis to explore their common pathogenesis, pathways, and immune‐related biomarkers. Meanwhile, three small molecule drugs, deguelin, berbamine, and osthole, also demonstrated promising binding affinities, offering the potential for development as targeted therapeutic drugs for glaucoma treatment. Overall, these results provide novel insights for future research on the immunopathogenesis of POAG and identify candidate diagnostic biomarkers for patients with SLE‐related POAG.

## 5 METHODS

### Study design and microarray data

The overall research design is summarized in Figure S5. POAG and SLE disease datasets were sourced from the gene expression omnibus (GEO) database. The search terms “POAG” and “SLE” were utilized, and the organisms and tissues were human trabecular meshwork tissue and blood, respectively. The POAG and SLE datasets GSE27276 and GSE50772, respectively, were included in this study. The GSE27276 data set contained 17 patients with POAG and 19 healthy controls. The GSE50772 data set included 61 patients with SLE and 20 healthy controls. The standard concrete can be found in the references [60, 61]. Single‐cell transcriptomic data of trabecular meshwork tissue from patients with glaucoma were also obtained from the GEO database (GSE148371), and the data contained six trabecular meshwork organizations of patients with POAG.

### Identification and visualization of DEGs

DEGs were analyzed utilizing the R package “Limma,” and original data were normalized by logarithmic transformation. Genes that satisfied the selection range (|logFC | > 0.7, adjusted *p* < 0.05) were considered DEGs. DEGs were visualized using R packages (pheatmap, dplyr, and ggplot2) to generate heatmaps and volcano plots. Venn diagrams were constructed employing the R package “Venn” to identify overlapping DEGs between POAG and SLE.

### Construction of gene co‐expression network

Genes exhibiting similar expression patterns were clustered using WGCNA to detect co‐expressed modules highly associated with the disease. First, a co‐expression network was built using an automated network system to calculate the correlation coefficients between genes, and a hierarchical clustering tree was constructed, leveraging these correlation coefficients as the basis for the intricate clustering of genes. Second, the correlation between the eigenvalues of the modules and disease phenotypes was calculated, and the modules with the highest correlation coefficients and most statistically significant *p*‐values were identified as pivotal gene modules [62].

### Gene correlation analysis

The PPI network was explored utilizing the STRING database (https://cn.string-db.org/). An interaction with a combined score exceeding 0.4 was selected and utilized to construct a PPI network with Cytoscape software (version 3.9.0). Additionally, the correlations between individual genes were computed based on the R package “corrplot.”

### Functional enrichment analysis

KEGG and GO enrichment analyses were conducted using the R packages “clusterProfiler” and “enrichplot.”

### Identification and validation of biomarkers

Two machine learning algorithms were employed to detect crucial biomarkers in patients with SLE‐related POAG: LASSO logistic regression and SVM‐RFE. LASSO logistic regression and SVM‐RFE were conducted utilizing the R packages “glmnet” and “e1071.” Data were evaluated based on receiver operating characteristic (ROC) curves, and the predictive efficacy of each algorithm was measured by calculating the AUC.

### Construction of TFs and miRNA regulatory networks of biomarkers

Regulatory networks of miRNAs and TFs were established using the NetworkAnalyst platform (https://www.networkanalyst.ca/), based on the miRTarBase v8.0 and JASPAR databases, respectively. The results were plotted using the Cytoscape software. By calculating the degree values, miRNAs and TFs were visualized with varying sizes based on their association strengths.

### Potential targeted drugs for prediction of biomarkers

Potentially effective drugs targeting biomarkers were anticipated utilizing the DSigDB database, and the top 10 drugs were selected for presentation based on *p*‐values. Subsequently, the three‐dimensional (3D) structures of proteins and small molecule drugs were separately obtained from Uniprot (https://www.uniprot.org/) and PubChem (https://pubchem.ncbi.nlm.nih.gov/), and Open Babel was used to convert small molecule drugs from SDF format to PDB format. Using AutoDock Vina (version 1.5.7) with a semi‐flexible docking approach, we conducted molecular docking of small molecule drugs and protein molecules. Each drug ligand and protein receptor were docked 50 times, and the binding energy was calculated. Finally, based on affinity and whether hydrogen bonds were formed, docking structures and active sites were determined and visualized using PyMOL (version 3.7.0) and Discovery Studio 2024 Client.

### Correlation analysis of two disease databases and biomarkers with immune cells

The relative influx abundance of immune cells in the two samples was assessed by ssGSEA enrichment analysis using the R package “GSVA,” and the disparity in the infiltration of SLE and POAG immune cells was observed. The correlation between characteristic biomarkers and immune cells was explored using CIBERSORT.

### Single‐cell transcriptome analysis

Data were processed using the R package “Seurat,” and the single‐cell data were clustered through tSNE analysis. Subsequently, marker genes were identified in each cluster and annotated to different cellular subtypes using the R software packages “celldex” and “SingleR.” Finally, violin and scatter plots were employed to visualize the distribution of hub genes.

### Cell culture

Human trabecular meshwork stem cells were purchased from IMMOCELL (Xiamen, China) and cultured at 37°C with 5% CO_2_ in Dulbecco's Modified Eagle Medium (DMEM)/F12 medium supplemented with 15% fetal bovine serum (FBS) (Gibco). When the cell density reached 80%, it was washed with PBS (Gibco) and treated with 0.05% trypsin (Gibco) for passage at a 1:3 ratio.

### H_2_O_2_ construction of a trabecular meshwork stem cell glaucoma model

H_2_O_2_ was procured from HUSHI. Trabecular meshwork stem cells were seeded with 5 × 10^5^ cells per well. The treatment group was cultivated in a medium supplemented with H_2_O_2_, while the control group was cultivated in DMEM/F12 medium at 37°C for 24 h to establish an in vitro glaucoma model and a control group, respectively [63].

### Cell viability assay

To assess cell viability, 5 × 10^3^ cells were seeded per well and partitioned into H_2_O_2_‐treated and control groups. The modeling group was treated with multiple concentrations of H_2_O_2_ (0.1, 10, 100, 200, 400, and 800 μM, and 1 mM), and four replicates of each group were set, cultured at 37°C for 24 h. Cell viability was evaluated utilizing cell counting kit‐8 (CCK‐8) (ECOTOP). The absorbance was recorded at 450 nm.

### RNA extraction and RT‐qPCR

TRIzol reagent was used to extract RNA from human trabecular meshwork stem cells, while cDNA was generated using a Hifair III cDNA Synthesis Kit (Yeasen). RT‐qPCR was conducted using QuantStudio 7 Flex with 2 × SYBR Green Master Mix (Yeasen). The primer sequences are listed in Table 1. The comparative computed tomography (CT) method (2^‐ΔΔCT^) was employed to ascertain the relative expression of genes, with untreated human trabecular meshwork stem cells serving as the control. The housekeeping gene glyceraldehyde‐3‐phosphate dehydrogenase (*GAPDH*) was utilized for gene expression normalization. The RT‐qPCR procedure primarily includes an initial denaturation at 95°C for 5 min, followed by denaturation at 95°C for 10 s, and annealing and extension at 60°C for a total of 30 s, for 40 cycles.

**Table 1:** Primer sequences.

Genes	Primer sequences
*MGP*	F GCCTGATCCTTCTTGCCATCCTG
	R AGCGTTCTCGGATCCTCTCTTGG
*FRZB*	F GCAGTAGTGGAGGTGAAGGAGATTC
	R GCCAGAGCTGGTATAGAGGTTGAC
*LAMB3*	F GCAGCCTCACAACTACTACAG
	R CCAGGTCTTACCGAAGTCTGA
*CSRNP1*	F ATAGGATCTGCGACCCTGAGAC
	R TGTGTGGTCCATCTGGCACTTG
*NFKBIA*	F ATGTGGACGACCGCCACGACA
	R ATGGCCAAGTGCAGGAACGAGTC
*GAPDH*	F ACAACTTTGGTATCGTGGAAGG
	R GCCATCACGCCACAGTTTC

### Statistical analysis

Bioinformatics data were statistically analyzed using R (version 4.3.1), and significance was determined at a *p*‐value of <0.05. CCK‐8 and RT‐qPCR data were visualized using Prism 9. Data were analyzed by one‐way analysis of variance (ANOVA) or Student's *t*‐test, and the results of ANOVA were analyzed by Dunnett's test. *p*‐values of <0.05 were deemed statistically significant. *p*‐values of <0.05, <0.01, <0.001, and <0.0001 are indicated by the symbols *, **, ***, and ****, respectively. Values represent the average of three independent experiments with three or four cell wells per treatment.

## AUTHOR CONTRIBUTIONS

**Yixian Liu**: Software; data curation; validation; visualization; writing—original draft; project administration. **Mengling You**: Methodology; validation. **Zhou Zeng**: Supervision. **Jing Wang**: Formal analysis; investigation. **Rong Rong**: Conceptualization; methodology; writing—review and editing; funding acquisition; project administration. **Xiaobo Xia**: Conceptualization; methodology; funding acquisition; writing—review and editing; resources.

## CONFLICT OF INTEREST STATEMENT

The authors declare no conflict of interest.

## ETHICS STATEMENT

No animals or humans were involved in this study.

## Supporting information


**Figure S1:** Differentially expressed genes (DEGs) in primary open‐angle glaucoma (POAG) and systemic lupus erythematosus (SLE) disease datasets. (A) Volcano plot of DEGs in POAG. (B) Volcano plot of DEGs in SLE. Red dots represent upregulated genes and blue dots represent downregulated genes.
**Figure S2:** Weighted gene co‐expression network analysis (WGCNA) of systemic lupus erythematosus (SLE) and primary open‐angle glaucoma (POAG) datasets. (A) Scatter plot describing gene salience (GS) and module members (MMs) in the module related to POAG. (B) Scatter plot describing GS and MMs in the module related to SLE.
**Figure S3:** Gene and pathway enrichment analysis of differentially expressed genes. (A) Bar graph of gene ontology (GO) enrichment analysis. (B) Bubble graph of Kyoto Encyclopedia of Genes and Genomes (KEGG) enrichment analysis.
**Figure S4:** Regulatory networks of hub genes. (A) microRNA (miRNA) regulatory network of biomarkers. (B) Transcription factor regulatory network of biomarkers.
**Figure S5:** Flowchart of the study design. The flow chart shows the steps of extracting samples from the primary open‐angle glaucoma (POAG) and systemic lupus erythematosus (SLE) datasets, performing weighted gene co‐expression network analysis (WGCNA) and differential expression analysis (DEA) analysis, finding the key genes, after enrichment analysis and PPI network analysis, and finally validating the diagnostic markers by reverse transcription quantitative real‐time polymerase chain reaction (RT‐qPCR), ROC curve, and single‐cell transcriptome sequencing.

**Table S1:** Gene expression matrix of glaucoma.
**Table S2:** Gene expression matrix of systemic lupus erythematosus.

## Data Availability

GSE50772, GSE27276, and GSE148371 were downloaded from the Gene Expression Omnibus (GEO) database (http://www.ncbi.nlm.nih.gov/geo/). The data and scripts used are saved in GitHub (https://github.com/Brooklyx/2024Liu). Supplementary materials (figures, tables, graphical abstracts, slides, videos, Chinese translated version, and updated materials) may be found in the online DOI or iMeta Science http://www.imeta.science/imetaomics/.
