# Supplementary material for: Novel insights into immunopathogenesis and crucial biomarkers between primary open‐angle glaucoma and systemic lupus erythematosus
Source: IMetaOmics. 2024 Sep 10;1(2):e27. doi: 10.1002/imo2.27 (PMC12806491; doi:10.1002/imo2.27)
Supplement: Supplementary file 1 — Figure S1: Differentially expressed genes (DEGs) in primary open‐angle glaucoma (POAG) and systemic lupus erythematosus (SLE) disease datasets. (A) Volcano plot of DEGs in POAG. (B) Volcano plot of DEGs in SLE. Red dots represent upregulated genes and blue dots represent downregulated genes. Figure S2: Weighted gene co‐expression network analysis (WGCNA) of systemic lupus erythematosus (SLE) and primary open‐angle glaucoma (POAG) datasets. (A) Scatter plot describing gene salience (GS) and module members (MMs) in the module related to POAG. (B) Scatter plot describing GS and MMs in the module related to SLE. Figure S3: Gene and pathway enrichment analysis of differentially expressed genes. (A) Bar graph of gene ontology (GO) enrichment analysis. (B) Bubble graph of Kyoto Encyclopedia of Genes and Genomes (KEGG) enrichment analysis. Figure S4: Regulatory networks of hub genes. (A) microRNA (miRNA) regulatory network of biomarkers. (B) Transcription factor regulatory network of biomarkers. Figure S5: Flowchart of the study design. The flow chart shows the steps of extracting samples from the primary open‐angle glaucoma (POAG) and systemic lupus erythematosus (SLE) datasets, performing weighted gene co‐expression network analysis (WGCNA) and differential expression analysis (DEA) analysis, finding the key genes, after enrichment analysis and PPI network analysis, and finally validating the diagnostic markers by reverse transcription quantitative real‐time polymerase chain reaction (RT‐qPCR), ROC curve, and single‐cell transcriptome sequencing. [file IMO2-1-e27-s001.docx]

**Supporting information to**

**Novel insights into immunopathogenesis and crucial biomarkers between primary open-angle glaucoma and systemic lupus erythematosus**

**Running title:** Comprehensive bioinformatics analysis of SLE and POAG

Yixian Liu^1,2,3,4#^, Mengling You^1,2,3,4#^, Zhou Zeng^1,2,3,4^, Jing Wang^1,2,3,4^, Rong Rong^1,2,3,4^* and Xiaobo Xia^1,2,3,4^*

1 Eye Center of Xiangya Hospital, Central South University, Changsha 410008, China

2 Hunan Key Laboratory of Ophthalmology, Changsha 410008, China

3 National clinical key specialty of ophthalmology, Changsha 410008, China

4 National Clinical Research Center for Geriatric Diseases (Xiangya Hospital), Central South University, Changsha 410008, China

#These authors contributed equally: Yixian Liu, Mengling You

*Correspondence: [xbxia21@csu.edu.cn](mailto:xbxia21@csu.edu.cn) [(Xiaobo Xia)](mailto:(Prof.%20Xiaobo%20Xia)) and rrong99@csu.edu.cn (Rong Rong)

**Supplementary Figures**

**Figure S1 DEGs in POAG and SLE disease datasets.** (A) Volcano plot of DEGs in POAG. (B) Volcano plot of DEGs in SLE. Red dots represent up-regulated genes and blue dots represent down-regulated genes.

**Figure S2 WGCNA of SLE and POAG datasets.** (A) Scatter plot describing gene salience (GS) and module members (MMs) in the module related to POAG. (B) Scatter plot describing GS and MMs in the module related to SLE.

**Figure S3 Gene and pathway enrichment analysis of differentially expression genes.** (A) Bar graph of GO enrichment analysis. (B) Bubble graph of KEGG enrichment analysis.

**Figure S4 Regulatory networks of hub genes.** (A) miRNA regulatory network of biomarkers. (B) TF regulatory network of biomarkers.

**Figure S5 Flowchart of the study design.** The flow chart shows the steps of extracting samples from the POAG and SLE datasets, performing WGCNA and DEA analysis, finding the key genes, after enrichment analysis and PPI network analysis, and finally validating the diagnostic markers by reverse transcription quantitative real-time polymerase chain reaction (RT-qPCR), ROC curve, and single-cell transcriptome sequencing.
